# Caregiver burden and perceived community support as correlates of family quality of life among caregivers of individuals with autism spectrum disorder in Saudi Arabia

**DOI:** 10.3389/fpsyg.2026.1897617

**Published:** 2026-09-30

**Authors:** Ghaniah Ain

**Affiliations:** Special Education Department, King Saud University, Riyadh, Saudi Arabia

**Keywords:** autism spectrum disorder, caregiver burden, family quality of life, Saudi Arabia, self-rated digital competence, service accessibility, perceived community support

## Abstract

Families raising a member with autism spectrum disorder face continuing psychological, financial, and logistical demands that can compromise family quality of life, yet Gulf-region evidence on the joint contribution of caregiver burden, perceived support, digital competence, and service accessibility remains scarce. This cross-sectional, exploratory study examined family quality of life and the incremental contribution of these factors among 294 caregivers with complete family-quality-of-life (FQoL) data, drawn from 416 survey respondents caring for an individual with autism spectrum disorder across the five administrative regions of Saudi Arabia between March and April 2026. Participants completed an Arabic questionnaire battery comprising a 25-item family quality of life measure structured on the five Beach Center domains but administered with frequency anchors, together with three measures developed for this study: caregiving difficulties, perceived support from caregiver and online communities, and self-rated digital competence, and a nine-item service accessibility composite. Family quality of life was moderate to good overall (mean = 3.77, standard deviation = 0.30). In hierarchical regression analyses, the final model accounted for 35.7% of the variance in family quality of life within this sample and under this specification. In the primary hierarchical regression model, perceived community support (standardized coefficient = 0.39), caregiver burden (−0.26), and the presence of a comorbid condition (−0.13) were independently associated with family quality of life; the comorbidity association, however, was less robust when professional caregivers were excluded. Self-rated digital competence, service accessibility, household income, educational attainment, and caregiver-reported autism severity were not independently associated with family quality of life once the other variables were included. Family quality of life differed between the mild and moderate caregiver-reported severity groups, but the study provides no reliable evidence concerning the severe group because only 10 participants (3.4%) were so classified, and severity carried no association in the adjusted model. Because the design was cross-sectional, all five composites were developed for this study and none is independently validated, two of them fall below conventional reliability thresholds, and the sample was a non-probability online sample, these findings are exploratory associations that require replication rather than estimates of determinants of family quality of life.

## 1 Introduction

Autism spectrum disorder (ASD) is a heterogeneous neurodevelopmental condition characterized by persistent deficits in social communication and by restricted, repetitive patterns of behavior, interests, or activities (American Psychiatric Association, 2022). The global prevalence of ASD has continued to rise, with the most recent estimates from the Centers for Disease Control and Prevention indicating that approximately 1 in 36 children in the United States meets diagnostic criteria (Maenner et al., 2023). In Saudi Arabia, national epidemiological data remain incomplete, although regional reports converge on prevalence estimates exceeding those of most neighboring countries (Alshaban et al., 2019; Alshaigi et al., 2020). Saudi Vision 2030 has positioned disability services as *a priori*ty area for system reform, yet structural and cultural barriers continue to shape the family experience of caregiving (Al Awaji et al., 2024; Saleh et al., 2024).

Caring for a family member with ASD entails sustained emotional, financial, and logistical demands, and existing evidence consistently documents reduced family wellbeing relative to families of typically developing children and, in some studies, relative to families raising children with other developmental disabilities (Hsiao et al., 2017; Mello et al., 2019; Vasilopoulou and Nisbet, 2016). Recent syntheses of families’ experiences of accessing intervention services further document substantial informational, financial, and geographic obstacles that compound these demands (Chow et al., 2024). Family quality of life (FQoL), conceptualized as the degree to which family needs are met, family members enjoy their lives together, and members have opportunities to pursue what is important to them (Park et al., 2003), has emerged as the most informative family-level outcome in autism research because it captures collective rather than individual functioning (Hoffman et al., 2006; Vasilopoulou and Nisbet, 2016).

A growing body of work has identified determinants of FQoL across multiple ecological strata. Caregiver burden—defined as the perceived physical, emotional, social, and financial strain arising from the ongoing provision of care to a dependent family member (Zarit et al., 1980)—is one of the most consistently reported correlates of reduced FQoL (Marsack-Topolewski and Church, 2019; Ortiz-Rubio et al., 2021). Perceived social support, defined as an individual’s appraisal of the availability and adequacy of emotional, instrumental, and informational assistance provided by family, friends, and significant others (Zimet et al., 1988), has shown a protective association with caregiver and family wellbeing in Saudi (Khusaifan and El Keshky, 2025) and international studies (Lai et al., 2015; Wang et al., 2021).

The role of digital resources has received increased attention in the post-pandemic period. Telehealth-delivered parent training and online peer networks have demonstrated feasibility and outcomes comparable to in-person formats for children with ASD (Kelson and Dorstyn, 2025; Pan et al., 2023). Digital health literacy—defined as the ability to seek, appraise, and apply electronic health information (Norman and Skinner, 2006)—has been proposed as an enabling condition for caregivers’ effective participation in such digital ecosystems (van der Vaart and Drossaert, 2017). Caregivers with higher digital health literacy report greater engagement with health information, more confident interaction with providers, and better self-rated outcomes (Knapp et al., 2011).

Service accessibility is defined in this study, following Penchansky and Thomas (1981), as the degree of fit between families and the service system across the dimensions of availability, geographic accessibility, accommodation including scheduling, affordability, and acceptability; it remains a persistent structural determinant of family outcomes (Mello et al., 2019). Saudi caregivers report dissatisfaction with service availability, long waiting lists, and high private-sector costs, with regional disparities particularly evident outside major urban centers (Al Awaji et al., 2024; Saleh et al., 2024). Comparable barriers have been documented in other health systems in Asia (Chow et al., 2024). The bioecological model (Bronfenbrenner, 1979) offers a useful organizing framework for these determinants: caregiver burden and self-rated digital competence operate at the microsystem level, perceived community support spans the meso- and exosystem and service accessibility reflects exo- and macrosystem influences. The proposed framework is illustrated in Figure 1.

**Figure 1:**
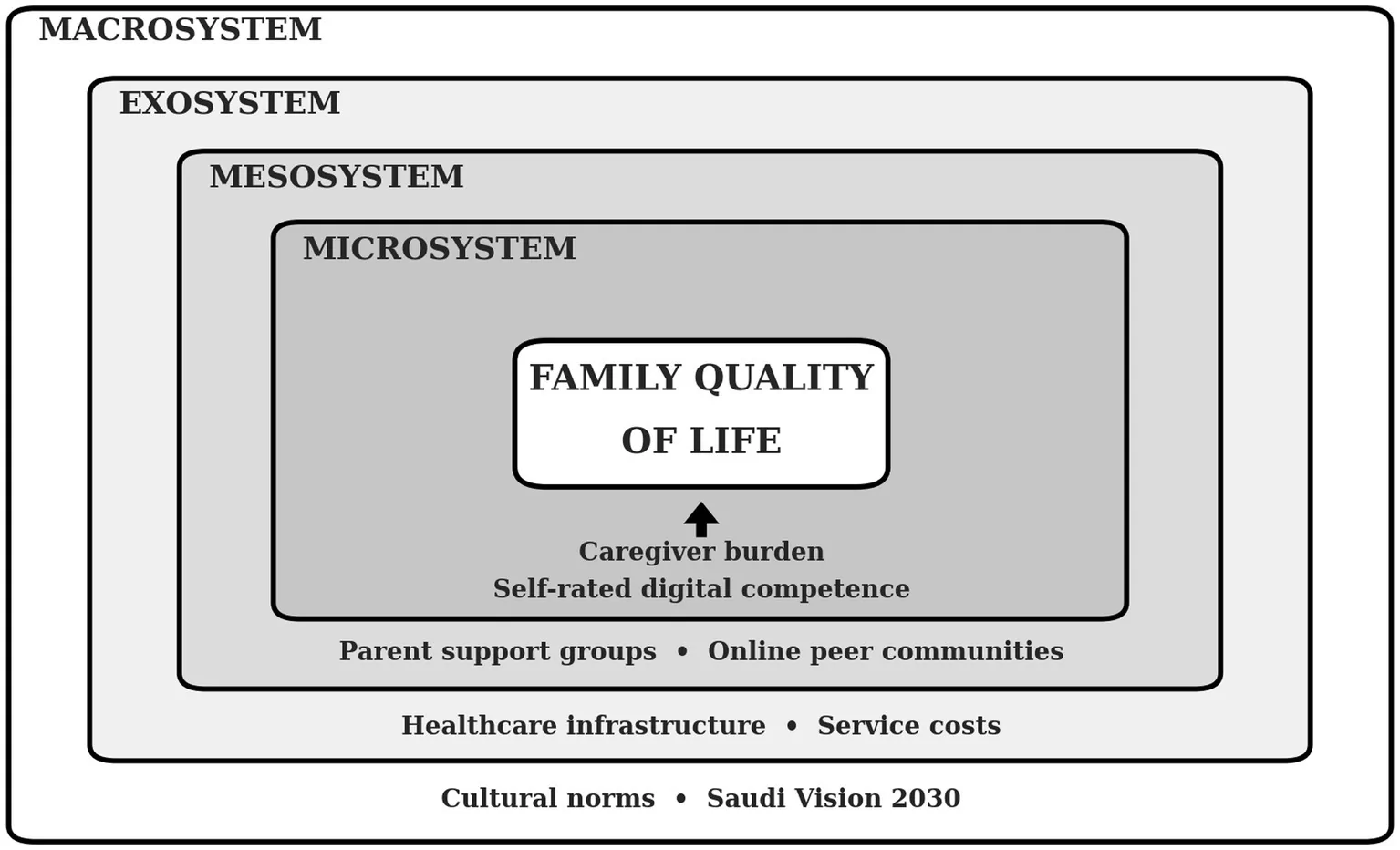
Bioecological framework organizing the correlates of family quality of life examined in this study (Bronfenbrenner, 1979). Caregiver burden and self-rated digital competence are located at the microsystem, parent support groups and online peer communities at the mesosystem, healthcare infrastructure and service costs at the exosystem, and cultural norms and Saudi Vision 2030 at the macrosystem.

Despite the relevance of these constructs, Gulf-region evidence examining their joint contribution to FQoL remains scarce. Existing Saudi studies have typically focused on single correlates, employed small or convenience-only samples, or relied on instruments without documented psychometric properties for the Arabic-speaking caregiver population (Al Mansoor, 2025; Saleh et al., 2024). Where Saudi work has examined caregiver outcomes directly, it has generally measured individual parental wellbeing, such as anxiety, depression, and personal quality of life, rather than functioning at the family level (Alenezi et al., 2024). The present study addresses these limitations in a sample of Saudi caregivers drawn from all five administrative regions. The aims were threefold: to quantify the level of FQoL and to describe levels of caregiver burden, perceived community support, self-rated digital competence, and service accessibility; to test, using a four-block hierarchical multiple regression, the incremental contribution of these constructs to explained variance in FQoL after accounting for clinical and family sociodemographic characteristics; and to identify the magnitude and direction of group differences in FQoL associated with ASD severity, comorbidity, and household income. Two terminological points apply throughout. First, the terms “predictor” and “predict” are used exclusively in their statistical sense, denoting variables entered into a regression model; given the cross-sectional design, no temporal or causal interpretation is implied. Second, the constructs reviewed above are the conceptual background to the study, but the instruments actually administered are narrower than those constructs, and the article names them accordingly. What is measured here as *perceived community support* is support reported from caregiver networks and online communities rather than general perceived social support in the sense of Zimet et al. (1988), and what is measured as *self-rated digital competence* is confidence in and engagement with digital environments rather than tested ability to seek, appraise, and apply health information in the sense of Norman and Skinner (2006). The study is accordingly exploratory, and its findings concern the composites it administered rather than the established constructs from which they take their inspiration.

## 2 Materials and methods

### 2.1 Design

A cross-sectional, correlational design was employed in accordance with the Strengthening the Reporting of Observational Studies in Epidemiology (STROBE) guidelines (von Elm et al., 2007). Data were collected between March and April 2026.

### 2.2 Participants and sampling

Inclusion criteria were being a primary caregiver of at least one family member with a formal medical diagnosis of ASD; being aged 18 years or older; residing in Saudi Arabia; and being able to read Arabic. The questionnaire was administered entirely as an electronic survey. A non-probability sampling strategy was used, in which the survey link was disseminated to the target population through autism-related associations and service providers, parent support groups and disability advocacy organizations, and verified social media channels, with an open invitation to eligible caregivers in all five administrative regions. Because the survey was self-administered and anonymous, and did not record the route by which each respondent reached the link, responses cannot be attributed to a particular dissemination channel.

A total of 416 caregivers submitted responses. Of these, 294 (70.7%) provided complete data on all measures entering the primary regression model and constitute the analytic sample. The 122 excluded responses were all excluded for the same reason: one or more items of the family quality of life measure were missing. No response was incomplete on the caregiving-difficulties, perceived-support, digital-competence, service-accessibility, or sociodemographic items, so listwise deletion was driven entirely by the outcome measure. Because 29.3% of submitted responses were lost in this way, and because the pattern of missingness on the outcome cannot be assumed to be unrelated to the outcome itself, the analytic sample may differ systematically from the full set of respondents; this is noted among the limitations. Participant flow is shown in Figure 2. Because a non-probability design was used, the representativeness of the sample relative to the national population of Saudi caregivers cannot be assumed; the implications of this are considered in Section 4.1.

**Figure 2:**
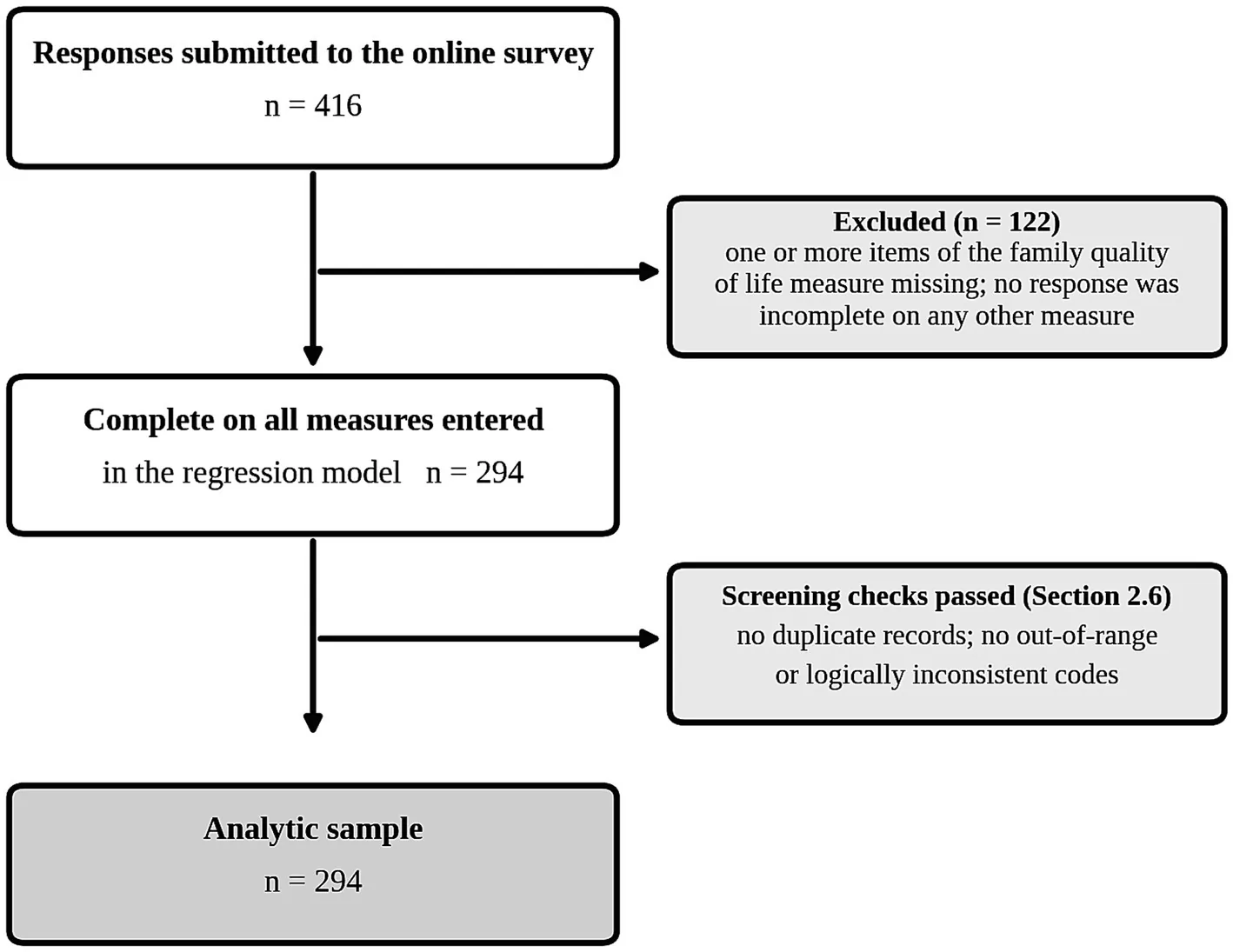
Participant flow from submitted responses to the analytic sample (*N* = 294). All 122 exclusions were for missing items on the family quality of life measure; no response was incomplete on any other measure. Screening checks are described in Section 2.6.

The sample is one of primary caregivers rather than parents specifically: alongside parents (66.3%) it includes siblings (15.6%), grandparents (9.2%), other relatives (5.4%), and a small number of professional caregivers (3.4%). The family member with ASD likewise spans a wide age range, from early childhood (0–5 years, 45.9%) through adolescence to young adulthood (18–25 years and above, 4.4%). All analyses were conducted on the full analytic sample; a sensitivity analysis excluding professional caregivers is reported in Section 3.6.

### 2.3 Sample size determination

An *a priori* power analysis was conducted using G*Power 3.1 (Faul et al., 2009) for multiple linear regression with eight predictors, *α* = 0.05, power = 0.95, and an assumed small-to-medium effect of *f*^2^ = 0.05; this specification requires 463 participants. The achieved sample of 294 falls substantially short of that target. At *N* = 294 the design retains approximately 0.78 power for *f*^2^ = 0.05 and reaches 0.95 power only for effects of *f*^2^ ≥ 0.09. Several coefficients in the final model are small and non-significant, and the study is not well placed to distinguish a genuinely null association from one it was underpowered to detect; non-significant results are therefore reported as inconclusive rather than as evidence of absence. No *post hoc* power calculation based on the observed effect size is reported, since observed power is a transformation of the *p* value and adds no information about the adequacy of the design (Hoenig and Heisey, 2001).

### 2.4 Measures

The battery was administered in Arabic. All five composites were study-specific: the FQoL measure, caregiving difficulties, perceived community support, self-rated digital competence, and service accessibility were all written for this study, and no published, independently validated instrument was administered. The service-accessibility items were informed by the Penchansky and Thomas (1981) access framework but were not taken from a published validated scale. Items were drafted by the research team from the domain definitions given in Section 1 and reviewed for clarity and relevance before fielding. Because no formal content-validity exercise, expert panel, forward–back translation, or independent validation study was carried out, and because no confirmatory factor analysis in an independent sample is available, the composites are reported here as study-specific exploratory indicators rather than as established measures of the constructs whose names they resemble. The consequences for interpretation are set out in Section 4.1. Published Arabic instruments do exist for several of these constructs, and their use in place of purpose-developed items would have been preferable; the decision not to use them constrains what the present findings can support.

Internal consistency is reported as Cronbach’s *α* and, because *α* assumes essential tau-equivalence, also as McDonald’s *ω* estimated from the first principal component. Both are reported in Table 1. Reliability of this kind speaks only to internal consistency and does not establish that a set of items is unidimensional or that it measures the construct named.

**Table 1:** Descriptive statistics and internal consistency for the primary study variables (*N* = 294).

Scale or subscale	Items	Anchors	*M*	*SD*	*α*	*ω*
Family quality of life (total)	25	1–5	3.77	0.30	0.74	0.80
Family interaction	6	1–5	3.80	0.35	0.27	0.56
Parenting	6	1–5	3.79	0.40	0.40	0.66
Emotional wellbeing	4	1–5	3.68	0.51	0.46	0.71
Physical/material wellbeing	5	1–5	3.79	0.43	0.38	0.64
Disability-related support	4	1–5	3.75	0.50	0.38	0.68
Caregiver burden	31	1–3	2.25	0.23	0.81	0.85
Perceived community support	44	1–3	2.37	0.19	0.83	0.86
Self-rated digital competence	35	1–3	2.38	0.15	0.66	0.73
Service accessibility	9	rescaled 1–5	3.69	0.45	0.66	0.77

*M*, arithmetic mean; *SD*, standard deviation; *α*, Cronbach’s alpha; *ω*, McDonald’s omega estimated from the first principal component. Composites are item means on the anchor metric shown. The service accessibility composite was formed as given in Equation 2. Subscale scores are reported for completeness but were not analyzed, because none attained acceptable internal consistency; only the FQoL total score is used. Both coefficients index internal consistency only; neither establishes unidimensionality or construct validity.

Response anchors differ across sections and are stated for each measure. All composites were computed as item means so that scores remain on the anchor metric, as given in Equation 1, where *x_ij_* is the response of participant *i* to item *j* and *k* is the number of items in the scale.

$${\bar{x}}_{i}=\frac{1}{k}\sum_{j=1}^{k}x_{\mathrm{ij}}$$ (1)

#### 2.4.1 Family quality of life

FQoL was assessed with a 25-item Arabic questionnaire developed for this study and structured on the five domains of the Beach Center Family Quality of Life Scale (Hoffman et al., 2006): Family Interaction (6 items), Parenting (6 items), Emotional Wellbeing (4 items), Physical/Material Wellbeing (5 items), and Disability-Related Support (4 items). Items were rated on a 5-point frequency scale from 1 (never) to 5 (very often). Because this anchor format differs from the satisfaction format of the published instrument, the measure is an adapted, frequency-based questionnaire rather than the Beach Center Family Quality of Life Scale itself; it is referred to throughout as the FQoL measure, and its scores are not comparable with studies using the published scale. This is acknowledged in Section 4.1. Internal consistency of the total score was acceptable (*α* = 0.74, *ω* = 0.80). The five subscales, however, were not reliable: *α* ranged from 0.27 (Family Interaction) to 0.46 (Emotional Wellbeing), with *ω* from 0.56 to 0.71 (Table 1). Subscale scores are therefore reported descriptively in Table 1 for completeness but are not analyzed, and no claim is made about the relative standing of the five domains. Only the total score is used in the analyses that follow.

#### 2.4.2 Caregiver burden

Caregiver burden was assessed with a 31-item purpose-developed battery of caregiving difficulties covering psychological and emotional strain (9 items), physical strain (3 items), personal and social restriction (7 items), occupational and financial strain (3 items), and difficulties associated with autism-related characteristics (9 items). Items were rated on a 3-point frequency scale from 1 (never) to 3 (always), with higher scores indicating greater burden. Internal consistency was good (*α* = 0.81, *ω* = 0.85).

#### 2.4.3 Perceived community support

Perceived community support was assessed with a 44-item purpose-developed battery covering general support receipt (5 items) and four domains of support obtained through caregiver networks and online communities: emotional support (10 items), companionship and belonging (10 items), informational support (10 items), and instrumental or administrative support (9 items). Items were rated on a 3-point frequency scale from 1 (never) to 3 (often). Internal consistency was good (*α* = 0.83, *ω* = 0.86). Because the item content concerns support obtained through caregiver networks and online communities rather than support from family, friends, and significant others generally, the composite is labeled perceived community support throughout and should not be read as an index of general perceived social support.

#### 2.4.4 Self-rated digital competence

Self-rated digital competence was assessed with a 35-item purpose-developed battery covering digital competence (6 items), digital safety and protection (5 items), digital coping (3 items), digital engagement in support and learning (9 items), adaptation to digital challenges (6 items), and confidence in and attitudes toward technology (6 items). Items were rated on a 3-point frequency scale from 1 (never) to 3 (always). Two negatively worded items (one on frustration when a child does not respond immediately, one on frustration when a technical difficulty arises) were reverse-scored before aggregation, so that a higher composite score indicates greater competence throughout. Internal consistency fell below the threshold adopted in Section 2.8 (*α* = 0.66, *ω* = 0.73), so this composite is the least reliable of those entering the model and its coefficient should be read as attenuated by measurement error. The items ask about confidence, engagement, and self-appraised skill; they do not test the ability to locate, appraise, and apply health information, and the composite is therefore labeled self-rated digital competence rather than digital health literacy.

A further six items assessed family recovery following adverse digital experiences. These items did not form a reliable composite (*α* = 0.24) and were therefore not analyzed as a scale; they are reported descriptively in the Supplementary material.

#### 2.4.5 Service accessibility

Service accessibility was assessed with nine items following the access framework of Penchansky and Thomas (1981): general availability of services in the respondent’s area (1 item, 5-point), ease of reaching specialists (1 item, 4-point), ease of obtaining a place or appointment for medical, diagnostic, educational or rehabilitative, consultative, and social services (5 items, 5-point), and satisfaction with government and with private services (2 items, 4-point). Because anchor counts differ across items, each item was rescaled to a proportion of its own range and the composite was then rescaled to a 1–5 metric, as given in Equation 2, where *m_j_* is the number of anchors of item *j*.

$$A_{i}=1+4\times \frac{1}{k}\sum_{j=1}^{k}\frac{x_{\mathrm{ij}}-1}{m_{j}-1}$$ (2)

Internal consistency fell below the threshold adopted in Section 2.8 (*α* = 0.66, *ω* = 0.77). Two further items recorded travel time to the nearest specialized medical facility and to the nearest educational or rehabilitative facility. These were retained as descriptive indicators rather than composite components, because including them reduced internal consistency further.

The dimensionality of the composite is uncertain, and the two criteria applied disagree. Exploratory principal-component analysis of the nine items in the analytic sample returned three components with eigenvalues above 1 (2.82, 1.11, and 1.08), the first accounting for 31.3% of item variance. Parallel analysis against the 95th percentile of eigenvalues from 1,000 random datasets of the same dimensions retained only the first component, the second and third observed eigenvalues falling below their random criteria (1.11 against 1.24; 1.08 against 1.17). Since the eigenvalue-greater-than-one rule is known to over-retain, the parallel-analysis result is the more defensible of the two, and we do not claim that the items are empirically multidimensional.

What can be said is narrower and still unfavorable. A single retained component accounting for only 31.3% of item variance is weak, and the unrotated loadings show that two items do not belong with the rest: general availability of services in the respondent’s area loads 0.00 on the first component and ease of reaching specialists loads −0.15, while the five appointment items and the two satisfaction items load between 0.50 and 0.74. The composite is thus dominated by appointment acquisition and satisfaction, with availability and geographic reach contributing almost nothing, even though these are distinct facets of access in the framework the items were written to reflect (Penchansky and Thomas, 1981). This analysis was exploratory and was not intended as a formal validation study; taken with the low internal consistency, it indicates that the composite should be interpreted with substantial caution. The full loadings and parallelanalysis results are provided in the Supplementary material. Averaging the nine items into a single score is therefore a simplification, and given both the low internal consistency and the marginal component structure, the composite is a weak measure. It is retained in this form only for comparability with the study’s *a priori* model, and no conclusion about service access should be drawn from its coefficient. Results involving service accessibility are interpreted with this in mind in Section 4.

#### 2.4.6 Sociodemographic and clinical variables

A purpose-developed form recorded caregiver role, age band, education, employment, household size and composition, region of residence, monthly household income, and characteristics of the family member with ASD including age band, sex, age at first diagnosis, severity, and comorbid conditions. ASD severity was recorded as mild, moderate, or severe as reported by the caregiver. Severity was not verified against clinical documentation and was not re-assessed with standardized observational instruments; the implications of this proxy measurement are considered in Section 4.1, and are particularly consequential here because only 10 participants (3.4%) reported severe ASD. Comorbidity was recorded as the presence of any co-occurring intellectual disability, health condition, or other condition.

### 2.5 Procedure

The survey opened with an information page describing the purpose of the study, the voluntary and anonymous nature of participation, and the right to withdraw at any point. Caregivers indicated informed consent electronically before any item was displayed, and those who did not consent were not shown the questionnaire. Consenting caregivers completed the battery in a single session on the online survey platform. No personally identifying information was collected, and all responses were stored on encrypted institutional servers.

### 2.6 Data screening

Before analysis the dataset was screened for missing values, out-of-range codes, logically inconsistent responses, and duplicate records. No out-of-range or logically inconsistent codes were found. Missingness was confined entirely to the items of the family quality of life measure, as described in Section 2.2 and shown in Figure 2.

Duplicate screening was carried out on the raw response fields as exported, before any recoding or aggregation. Each record was compared with every other record by exact matching across all 192 questionnaire columns simultaneously, including the free-text fields; the export contains no platform-generated metadata columns, so no fields were excluded from the comparison. Treating missing values as matching, all 416 submitted responses are distinct, and all 294 analytic records are distinct; no response duplicates another. Because the survey platform did not record submission timestamps or respondent identifiers, independence of responses cannot be verified beyond this check, and the possibility that one respondent completed the survey more than once with different answers cannot be excluded.

### 2.7 Ethical considerations

The study received ethical approval from the Institutional Review Board of King Saud University (approval no. KSU-HE-26-0431). Informed electronic consent was obtained from all participants before the questionnaire commenced. Participation was voluntary and anonymous and could be discontinued at any point without consequence.

### 2.8 Data analysis

Analyses were conducted in Python 3.13 using pandas (McKinney, 2010), NumPy (Harris et al., 2020), SciPy (Virtanen et al., 2020), statsmodels (Seabold and Perktold, 2010), and scikit-posthocs (Terpilowski, 2019). Arabic response labels were normalized for orthographic variants and mapped to ordered numeric codes; no data values were altered. Internal consistency was estimated with Cronbach’s *α*, applying *α* ≥ 0.70 as the threshold for acceptability (Nunnally and Bernstein, 1994). Distributional normality was examined through skewness, kurtosis, and the Shapiro–Wilk test (Shapiro and Wilk, 1965); all five composites departed significantly from normality, and non-parametric procedures were therefore used for bivariate and group comparisons. Spearman rank-order correlations were used for bivariate associations, with the Benjamini–Hochberg false discovery rate procedure (Benjamini and Hochberg, 1995) applied across the family of tests.

Group differences in FQoL were tested with Mann–Whitney *U* and Kruskal–Wallis *H* tests. Effect sizes were the rank-biserial correlation for two-group comparisons and epsilon-squared for multi-group comparisons, computed as given in Equations 3 and 4, where *n*_1_ and *n*_2_ are the group sizes and *N* is the analytic sample size.

$$r_{\mathrm{rb}}=1-\frac{2U}{n_{1}n_{2}}$$ (3)

$$\varepsilon^{2}=\frac{H}{N-1}$$ (4)

Significant Kruskal–Wallis tests were followed by Dunn’s pairwise comparisons with Holm correction (Dunn, 1964).

Categorical variables were coded as follows. Comorbidity was coded dichotomously (0 = none, 1 = present). ASD severity (1 = mild, 2 = moderate, 3 = severe), monthly household income (1 = <SAR 5,000 to 4 = SAR 15,000–19,999), and caregiver educational attainment (1 = below secondary, 2 = bachelor, 3 = postgraduate) were entered as single ordinal scores. Together with caregiver burden, perceived community support, self-rated digital competence, and service accessibility, this yielded eight predictors. Because ordinal entry imposes an equal-interval assumption that is unlikely to hold exactly, a sensitivity analysis was conducted in which severity, income, and education were dummy coded (reference categories: mild, <SAR 5,000, and below secondary), producing a 12-predictor model.

A four-block hierarchical multiple regression predicted FQoL. The entry order was specified *a priori* on theoretical grounds, following a distal-to-proximal and least-to-most-modifiable sequence consistent with the bioecological framing in Figure 1: Block 1 entered fixed clinical characteristics (ASD severity and comorbidity), which are temporally antecedent to family adaptation and not modifiable through psychosocial intervention; Block 2 added the family’s relatively stable structural socioeconomic position (household income and educational attainment); Block 3 added caregiver burden as the most proximal, appraisal-level variable; and Block 4 entered the three resource variables (perceived community support, self-rated digital competence, and service accessibility) last, so that their increment to explained variance was estimated conservatively. The change in *F* associated with each block was computed as given in Equation 5, where *k* is the number of predictors after the block, *p* the number before it, and *N* the sample size.

$$\mathrm{\Delta F}=\frac{\left(R_{k}^{2}-R_{p}^{2})/(k-p\right)}{\left(1-R_{k}^{2})/(N-k-1\right)}$$ (5)

Regression assumptions were evaluated on the final model: linearity and homoscedasticity through plots of standardized residuals against fitted values and the Breusch–Pagan test (Breusch and Pagan, 1979); normality of residuals through quantile–quantile plots, skewness, kurtosis, and the Shapiro–Wilk test; independence of errors through the Durbin–Watson statistic; and influential observations through Cook’s distance. Because the Breusch–Pagan test indicated heteroscedasticity, heteroscedasticity-consistent (HC3) standard errors are reported alongside ordinary least squares estimates, and inference is based on the robust errors. Variance inflation factors were inspected with VIF ≤ 5 as the threshold (Field, 2018). The unique contribution of each predictor was quantified by the squared semipartial correlation, computed as given in Equation 6, where *t_i_* is the *t* statistic of predictor *i* and *df*_res_ the residual degrees of freedom of the final model.

$$sr_{i}^{2}=\frac{t_{i}^{2}(1-R^{2})}{df_{\mathrm{res}}}$$ (6)

Because the outcome is a bounded item mean on a 1–5 scale and several composites are skewed, four checks on the adequacy of the linear model were carried out in addition to the assumption tests above. Floor and ceiling occupancy was computed for every composite. Linearity was examined through the Ramsey RESET test with squared and cubed fitted values and through mean FQoL by tertile of each continuous predictor. Coefficient stability was assessed by three alternative estimators applied to the final model: percentile bootstrap confidence intervals from 5,000 resamples, Huber M-estimation, and median (quantile) regression. These serve as sensitivity analyses; the linear model estimated by ordinary least squares with HC3 standard errors remains the reported specification, and the alternatives are used to establish whether its conclusions depend on distributional assumptions.

Two further sensitivity analyses re-estimated the final model on modified samples—excluding the lowest income band and excluding professional caregivers—alongside the dummy-coded specification. Results are reported in Section 3.6 and Tables 2 and 3.

**Table 2:** Full dummy-coded sensitivity model predicting total family quality of life (*N* = 294).

Term	*B*	SE_r_	95% CI	*t*	*p*
Comorbidity (present)	−0.069	0.032	−0.133, −0.006	−2.14	0.033
Caregiver burden	−0.361	0.066	−0.491, −0.231	−5.46	<0.001
Perceived community support	0.605	0.092	0.424, 0.786	6.59	<0.001
Self-rated digital competence	0.181	0.103	−0.023, 0.384	1.75	0.081
Service accessibility	0.032	0.033	−0.034, 0.097	0.96	0.339
Severity: moderate v. mild	0.056	0.029	−0.001, 0.113	1.93	0.055
Severity: severe v. mild	−0.087	0.082	−0.248, 0.075	−1.05	0.293
Income: 5,000–9,999 v. <5,000	0.041	0.104	−0.165, 0.246	0.39	0.697
Income: 10,000–14,999 v. <5,000	0.033	0.104	−0.171, 0.238	0.32	0.748
Income: 15,000–19,999 v. <5,000	−0.033	0.106	−0.242, 0.177	−0.31	0.758
Education: bachelor v. below secondary	−0.068	0.083	−0.231, 0.094	−0.82	0.410
Education: postgraduate v. below secondary	−0.051	0.092	−0.231, 0.130	−0.55	0.581

Dependent variable: family quality of life total score. Model: *F*(12, 281) = 13.98, *p* < 0.001, *R*^2^ = 0.374, adjusted *R*^2^ = 0.347. *B*, unstandardized coefficient; SE_r_, heteroscedasticity-consistent (HC3) standard error; 95% CI, HC3 confidence interval for *B*; *t* and *p* computed from the HC3 errors. Reference categories: mild severity, income <SAR 5,000, education below secondary. The ordinary least squares *p* values agree with the HC3 values on every term.

**Table 3:** Robustness of the final-model coefficients across estimators and samples.

Predictor	OLS *B*	HC3 95% CI	Bootstrap 95% CI	Huber *B*	Median *B*
ASD severity	0.022	−0.031, 0.074	−0.028, 0.074	0.018	0.020
Comorbidity (present)	−0.075	−0.137, −0.013	−0.136, −0.013	−0.085	−0.095
Household income	−0.031	−0.069, 0.007	−0.069, 0.006	−0.034	−0.028
Educational attainment	0.001	−0.071, 0.073	−0.071, 0.070	0.009	0.050
Caregiver burden	−0.345	−0.474, −0.217	−0.475, −0.224	−0.296	−0.279
Perceived community support	0.623	0.445, 0.801	0.448, 0.800	0.567	0.587
Self-rated digital competence	0.169	−0.031, 0.369	−0.015, 0.377	0.141	0.134
Service accessibility	0.028	−0.038, 0.093	−0.036, 0.094	0.038	0.032

Dependent variable: family quality of life total score. OLS, ordinary least squares on the analytic sample (*N* = 294); HC3, heteroscedasticity-consistent interval; Bootstrap, percentile interval from 5,000 resamples; Huber, M-estimation with Huber weighting; Median, median (quantile) regression, pseudo *R*^2^ = 0.210. All four specifications identify the same three predictors as significant. Two further sensitivity models are reported in Section 3.6: excluding the lowest income band (*n* = 286, *R*^2^ = 0.362) and excluding professional caregivers (*n* = 284, *R*^2^ = 0.361); in the latter the comorbidity coefficient falls to *B* = −0.061, *p* = 0.056.

Effect-size conventions followed Cohen (1988). The significance level was set at *p* < 0.05 (two-tailed). Given the number of models reported, the absence of a pre-registered analysis plan, and the exploratory status of the measures, all inferential results are treated as exploratory.

## 3 Results

### 3.1 Sample characteristics

Of 416 caregivers who submitted responses, 294 provided complete data for the primary analyses. Parents comprised the largest proportion of caregivers (66.3%), followed by siblings (15.6%), grandparents (9.2%), other relatives (5.4%), and professional caregivers (3.4%). The modal caregiver age band was 36–45 years (47.3%). Most caregivers held a bachelor’s degree (79.9%) and reported a monthly household income of SAR 10,000–14,999 (37.4%) or SAR 15,000–19,999 (33.3%). The Western region contributed the largest share of participants (36.7%), followed by the Central region (22.8%). Family members with ASD were slightly more often male (54.1%), with a modal age band of 0–5 years (45.9%); severity was reported as mild (55.8%), moderate (40.8%), or severe (3.4%), and 45.2% had at least one comorbid condition. Most caregivers reported travel times of 1–2 h to the nearest specialized medical facility (64.6%) and to the nearest educational or rehabilitative facility (66.0%). Sample characteristics are summarized in Table 4.

**Table 4:** Sociodemographic and clinical characteristics of the analytic sample (*N* = 294).

Characteristic	Category	*n*	%
Caregiver role	Parent	195	66.3
	Sibling	46	15.6
	Grandparent	27	9.2
	Other relative	16	5.4
	Professional caregiver	10	3.4
Caregiver age band	<25 years	15	5.1
	25–35 years	53	18.0
	36–45 years	139	47.3
	46–55 years	63	21.4
	>55 years	24	8.2
Educational attainment	Below secondary	8	2.7
	Bachelor	235	79.9
	Postgraduate	51	17.3
Employment	Private-sector employee	131	44.6
	Public-sector employee	97	33.0
	Self-employed	43	14.6
	Retired	20	6.8
	Not employed	3	1.0
Monthly household income (SAR)	<5,000	8	2.7
	5,000–9,999	78	26.5
	10,000–14,999	110	37.4
	15,000–19,999	98	33.3
Region of residence	Central	67	22.8
	Western	108	36.7
	Eastern	53	18.0
	Northern	41	13.9
	Southern	25	8.5
Sex of family member with ASD	Male	159	54.1
	Female	135	45.9
Age band of family member	Early childhood (0–5 years)	135	45.9
	Middle childhood (6–12 years)	100	34.0
	Adolescence (13–18 years)	33	11.2
	Young adult (18–25+ years)	13	4.4
	Not reported	13	4.4
Age at first diagnosis	<2 years	96	32.7
	2–4 years	109	37.1
	5–6 years	61	20.7
	6–9 years	22	7.5
	Not recalled	6	2.0
ASD severity	Mild	164	55.8
	Moderate	120	40.8
	Severe	10	3.4
Comorbid condition	None	161	54.8
	Intellectual disability	90	30.6
	Health condition	38	12.9
	Other	5	1.7

SAR, Saudi Arabian Riyals; ASD, autism spectrum disorder. ASD severity was caregiver-reported. Percentages may not sum to 100 due to rounding.

Because 122 responses were excluded for missing outcome data, the analytic sample was compared with the excluded responses on every variable available for both groups. The four composite predictors were complete for all 416 respondents, as were the sociodemographic and clinical items, so the comparison covers caregiver burden, perceived community support, self-rated digital competence, service accessibility, caregiver role, educational attainment, household income, region of residence, ASD severity, and comorbidity. Every one of these variables was complete for all 416 respondents, so each comparison uses the full 294 against 122 split. No difference reached significance on any of the 10 variables. The Mann–Whitney comparisons of the four composites returned *p* values from 0.100 to 0.915, with effect sizes of *r* = 0.01 to 0.08 and bootstrap confidence intervals whose upper bounds did not exceed 0.18. The six chi-square tests returned *p* values from 0.103 to 0.827, with Cramér’s *V* from 0.03 to 0.14 and upper confidence bounds not exceeding 0.24. Supplementary Table S1 reports the sample size, test statistic, exact *p* value, effect size, and bootstrap confidence interval for each comparison. This does not establish that the data are missing completely at random, since missingness is defined on the outcome and the outcome is by definition unobserved in the excluded group, but it provides no evidence that the analytic sample differs from the excluded respondents on any measured characteristic.

### 3.2 Descriptive statistics for primary variables

FQoL was rated in the moderate-to-good range (*M* = 3.77, *SD* = 0.30 on the 1–5 metric). Caregiver burden was moderate (*M* = 2.25, *SD* = 0.23 on the 1–3 metric), as were perceived community support (*M* = 2.37, *SD* = 0.19) and self-rated digital competence (*M* = 2.38, *SD* = 0.15). Service accessibility was rated favorably (*M* = 3.69, *SD* = 0.45 on the rescaled 1–5 metric), above the scale midpoint of 3. Descriptive statistics and reliability coefficients are presented in Table 1. As set out in Section 2.4.1, the FQoL subscales did not attain acceptable internal consistency and are not analyzed; the subscale means in Table 1 are given for completeness only.

The dispersion of every composite is narrow. The FQoL total ranges from 2.52 to 4.28 on a 1–5 scale with a standard deviation of 0.30, and the three-point composites span less than one scale point each. FQoL and service accessibility departed significantly from normality (Shapiro–Wilk *p* < 0.001 in both cases, with skewness of −1.07 and −0.92 respectively); caregiver burden and perceived community support departed marginally (*p* = 0.032 and *p* = 0.038) and self-rated digital competence did not (*p* = 0.167). Non-parametric procedures were used for all bivariate and group comparisons.

### 3.3 Bivariate associations

FQoL total scores were positively associated with perceived community support (*ρ* = 0.468, *p* < 0.001) and self-rated digital competence (*ρ* = 0.310, *p* < 0.001) and negatively associated with caregiver burden (*ρ* = −0.285, *p* < 0.001). Service accessibility was not associated with FQoL (*ρ* = −0.013, *p* = 0.825). All three significant associations survived Benjamini–Hochberg correction.

Perceived community support and self-rated digital competence were strongly intercorrelated (*ρ* = 0.565, *p* < 0.001), which is expected given that both concern engagement with digital environments. Caregiver burden was not significantly associated with either support (*ρ* = 0.063, *p* = 0.278) or digital competence (*ρ* = 0.020, *p* = 0.734), nor with accessibility (*ρ* = −0.004). Correlations among the primary variables are reported in Table 5, and the full matrix including the FQoL subscales is displayed in Figure 3.

**Table 5:** Spearman rank-order correlations among the primary study variables (*N* = 294).

Variable	1	2	3	4	5
1. Family quality of life	–				
2. Caregiver burden	−0.285^***^	–			
3. Perceived community support	0.468^***^	0.063	–		
4. Self-rated digital competence	0.310^***^	0.020	0.565^***^	–	
5. Service accessibility	−0.013	−0.004	−0.075	−0.131^*^	–

Values are Spearman’s *ρ*. The Benjamini–Hochberg false discovery rate correction was applied across the correlation family. ^*^p < 0.05; ^***^p < 0.001.

**Figure 3:**
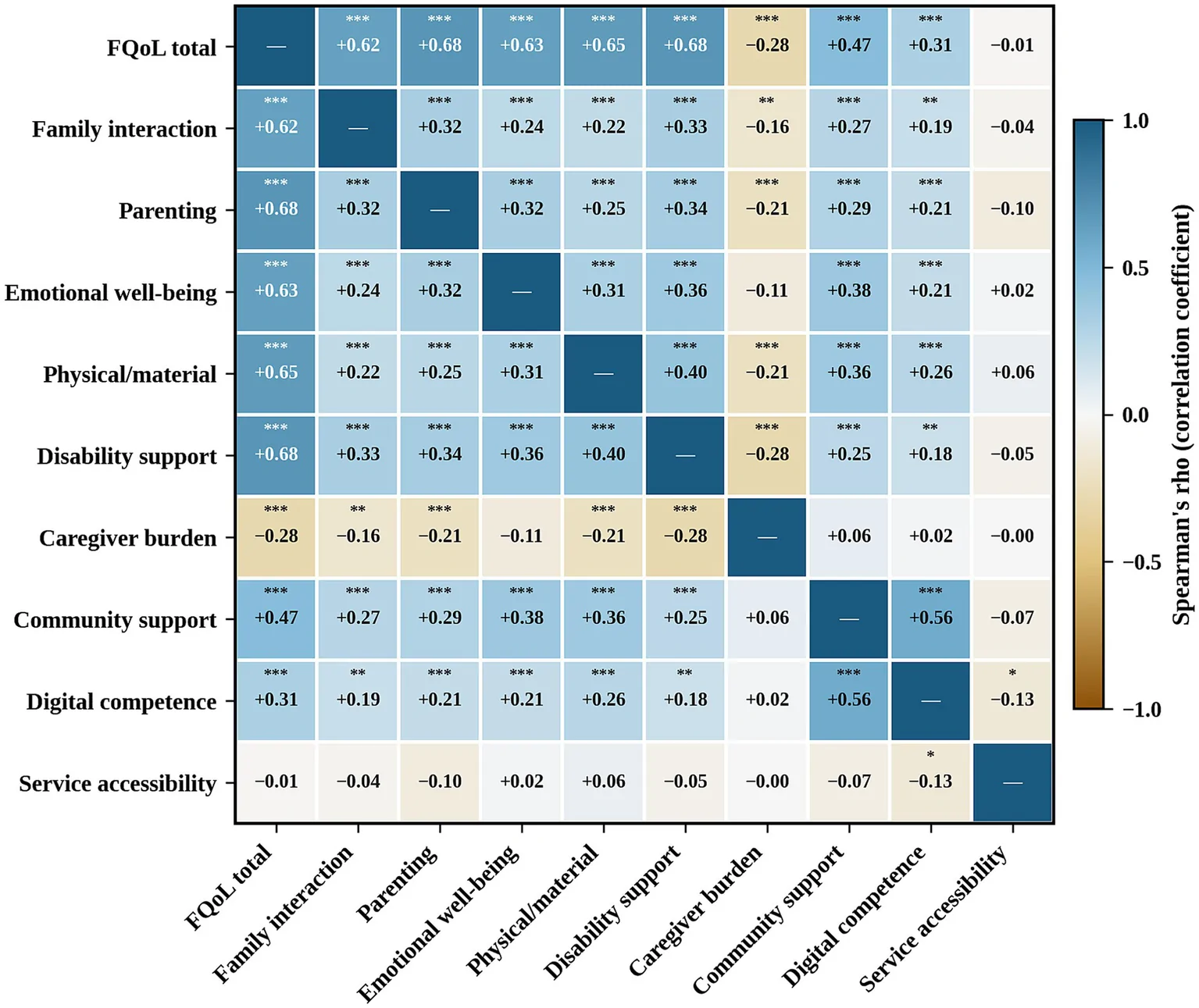
Spearman rank-order correlation matrix for total family quality of life, its five subscales, and the four primary correlates (*N* = 294). Cell values are correlation coefficients; shading indicates the sign and magnitude of the coefficient as shown on the scale bar. Asterisks denote significance after Benjamini–Hochberg false discovery rate correction: ^*^p < 0.05; ^**^p < 0.01; ^***^p < 0.001. Subscale correlations are shown for completeness; the subscales were not analyzed because none attained acceptable internal consistency (Table 1).

### 3.4 Group differences in family quality of life

Group differences in FQoL by ASD severity were significant but not ordered, *H*(2) = 13.70, *p* = 0.001, *ε*^2^ = 0.047: mean scores were 3.72 (*SD* = 0.31) in the mild group, 3.84 (*SD* = 0.27) in the moderate group, and 3.69 (*SD* = 0.30) in the severe group. Dunn’s pairwise comparisons with Holm correction located the effect entirely in the mild-versus-moderate contrast (*z* = −3.55, adjusted *p* = 0.001); neither contrast involving the severe group approached significance (mild versus severe, *z* = 0.47, adjusted *p* = 0.640; moderate versus severe, *z* = 1.76, adjusted *p* = 0.157), which is unsurprising given that only 10 participants reported severe ASD. The pattern is non-monotonic and does not describe a gradient of FQoL across severity.

Caregivers of family members with at least one comorbid condition reported lower FQoL (*M* = 3.67, *SD* = 0.31) than those without comorbidities (*M* = 3.85, *SD* = 0.26), *U* = 14625.5, *p* < 0.001, *r*_rb_ = 0.37, a medium effect by the conventions of Cohen (1988).

Household income groups differed, *H*(3) = 31.91, *p* < 0.001, *ε*^2^ = 0.109, with FQoL declining across bands from *M* = 3.89 (*SD* = 0.24) in the <SAR 5,000 group to *M* = 3.65 (*SD* = 0.31) in the SAR 15,000–19,999 group. Dunn’s comparisons with Holm correction showed that the highest band differed from the two middle bands (*z* = 5.17 and 4.12, adjusted *p* < 0.001), while no other contrast reached significance, including every contrast involving the lowest band, which contains only eight participants. The lowest band was inspected at the record level: its eight records are distinct, their FQoL scores range from 3.48 to 4.12 with a standard deviation of 0.244, and one of the eight reports a comorbid condition. The band shows no anomaly, but with *n* = 8 its mean is imprecise.

Educational attainment was not associated with FQoL, *H*(2) = 2.34, *p* = 0.311, *ε*^2^ = 0.008, nor was the sex of the family member with ASD, *U* = 11212.0, *p* = 0.509, *r*_rb_ = 0.04. Region of residence was associated with FQoL, *H*(4) = 13.62, *p* = 0.009, *ε*^2^ = 0.046, with the Northern (*M* = 3.89) and Southern (*M* = 3.87) regions highest and the Eastern region lowest (*M* = 3.71). Group comparisons are summarized in Table 6.

**Table 6:** Group differences in total family quality of life (*N* = 294).

Grouping variable	Group	*n*	*M* (*SD*)	Test	Statistic	*p*	Effect size
ASD severity	Mild	164	3.72 (0.31)	*H*(2)	13.70	0.001	*ε*^2^ = 0.047
	Moderate	120	3.84 (0.27)				
	Severe	10	3.69 (0.30)				
Comorbid condition	None	161	3.85 (0.26)	*U*	14625.5	<0.001	*r*_rb_ = 0.37
	Present	133	3.67 (0.31)				
Household income (SAR)	<5,000	8	3.89 (0.24)	*H*(3)	31.91	<0.001	*ε*^2^ = 0.109
	5,000–9,999	78	3.86 (0.25)				
	10,000–14,999	110	3.80 (0.29)				
	15,000–19,999	98	3.65 (0.31)				
Educational attainment	Below secondary	8	3.81 (0.35)	*H*(2)	2.34	0.311	*ε*^2^ = 0.008
	Bachelor	235	3.78 (0.29)				
	Postgraduate	51	3.72 (0.31)				
Sex of family member	Female	135	3.78 (0.30)	*U*	11212.0	0.509	*r*_rb_ = 0.04
	Male	159	3.76 (0.30)				
Region of residence	Central	67	3.76 (0.31)	*H*(4)	13.62	0.009	*ε*^2^ = 0.046
	Western	108	3.73 (0.31)				
	Eastern	53	3.71 (0.33)				
	Northern	41	3.89 (0.21)				
	Southern	25	3.87 (0.22)				

*U*, Mann–Whitney *U*; *H*, Kruskal–Wallis *H*; *r*_rb_, rank-biserial correlation (Equation 3); *ε*^2^, epsilon-squared (Equation 4); SAR, Saudi Arabian Riyals. Effect-size benchmarks for *r*_rb_ follow Cohen (1988): small ≥ 0.10, medium ≥ 0.30, large ≥ 0.50. Significant *H* tests were followed by Dunn’s pairwise comparisons with Holm correction, reported in Section 3.4.

### 3.5 Hierarchical multiple regression

Block 1, which entered ASD severity and the presence of comorbid conditions, explained 10.2% of the variance in FQoL, Δ*F*(2, 291) = 16.49, *p* < 0.001. Adding household income and educational attainment in Block 2 produced a small increment (Δ*R*^2^ = 0.035; Δ*F*(2, 289) = 5.89, *p* = 0.003). Caregiver burden, entered in Block 3, accounted for a further 3.5% (Δ*R*^2^ = 0.035; Δ*F*(1, 288) = 12.24, *p* < 0.001). Block 4, which added perceived community support, self-rated digital competence, and service accessibility, contributed 18.5% (Δ*R*^2^ = 0.185; Δ*F*(3, 285) = 27.31, *p* < 0.001)—the largest single increment. The final model accounted for 35.7% of the variance in FQoL (adjusted *R*^2^ = 0.339; *F*(8, 285) = 19.78, *p* < 0.001). Block-level results are presented in Table 7 and illustrated in Figure 4.

**Table 7:** Hierarchical multiple regression predicting total family quality of life (*N* = 294).

Block	Predictors entered	*R* ^2^	Adj. *R*^2^	Δ*R*^2^	Δ*F* (*df*)	*p*
1	ASD severity, comorbidity	0.102	0.096	0.102	16.49 (2, 291)	<0.001
2	+ Income, education	0.137	0.125	0.035	5.89 (2, 289)	0.003
3	+ Caregiver burden	0.172	0.158	0.035	12.24 (1, 288)	<0.001
4	+ Community support, digital competence, accessibility	0.357	0.339	0.185	27.31 (3, 285)	<0.001

Dependent variable: family quality of life total score. Δ*F*, change in *F* associated with the predictors entered at each block, computed as given in Equation 5. Overall final model: *F*(8, 285) = 19.78, *p* < 0.001; adjusted *R*^2^ = 0.339. Variance inflation factors for the final model ranged from 1.06 to 1.56.

**Figure 4:**
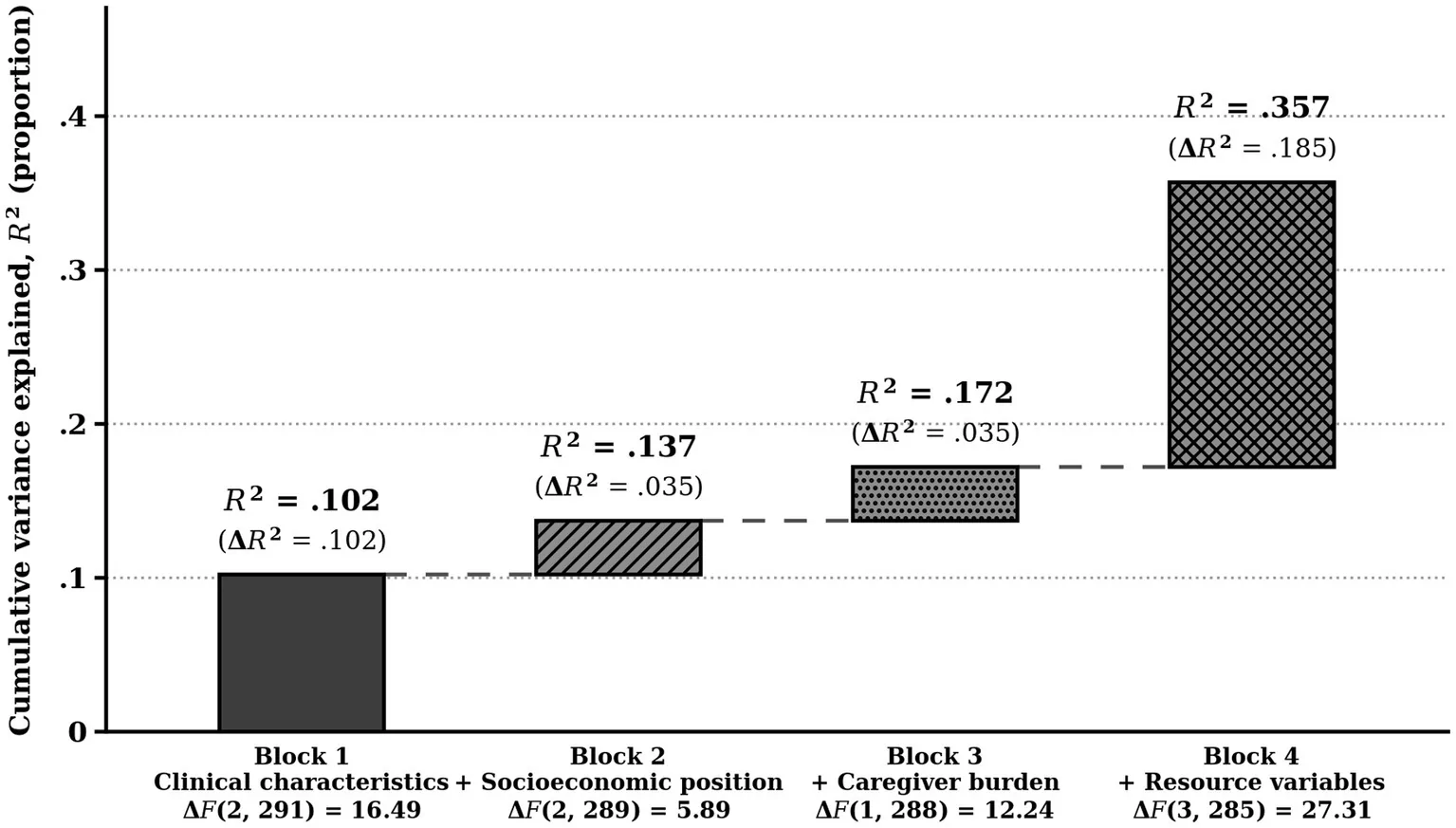
Cumulative proportion of variance in family quality of life explained across the four blocks of the hierarchical regression model (*N* = 294). Bar height gives the increment contributed by each block; the dashed connectors and the labels above each bar give the cumulative *R*^2^. The change in *F* and its degrees of freedom are given beneath each block label. All increments were significant at *p* < 0.01.

Only three of the eight predictors were independently associated with FQoL under the HC3 standard errors on which inference is based. Perceived community support showed the strongest association (*β* = 0.394, *p* < 0.001), followed by caregiver burden (*β* = −0.264, *p* < 0.001) and the presence of a comorbid condition (*β* = −0.125, *p* = 0.019). Self-rated digital competence did not reach significance (*β* = 0.084, *p* = 0.098), and neither did household income (*p* = 0.110), ASD severity (*p* = 0.412), service accessibility (*p* = 0.409), or educational attainment (*p* = 0.974). Notably, the whole of the Block 4 increment is attributable to perceived community support: digital competence and accessibility added nothing individually. Variance inflation factors ranged from 1.06 to 1.56, indicating no problematic multicollinearity. Full coefficients are presented in Table 8.

**Table 8:** Coefficients for the final hierarchical regression model predicting total family quality of life (*N* = 294).

Predictor	*B*	SE	*β*	95% CI	*t*	*p*	sr^2^	SE_r_	*p*_r_
ASD severity	0.022	0.026	0.041	−0.03, 0.07	0.83	0.409	0.002	0.027	0.412
Comorbidity	−0.075	0.031	−0.125	−0.14, −0.01	−2.43	0.016	0.013	0.032	0.019
Household income	−0.031	0.020	−0.087	−0.07, 0.01	−1.57	0.118	0.006	0.019	0.110
Education	0.001	0.034	0.002	−0.07, 0.07	0.04	0.972	0.000	0.037	0.974
Caregiver burden	−0.345	0.067	−0.264	−0.48, −0.21	−5.12	<0.001	0.059	0.065	<0.001
Community support	0.623	0.093	0.394	0.44, 0.81	6.70	<0.001	0.101	0.090	<0.001
Digital competence	0.169	0.119	0.084	−0.07, 0.40	1.42	0.157	0.005	0.102	0.098
Service accessibility	0.028	0.034	0.041	−0.04, 0.09	0.82	0.412	0.002	0.033	0.409

Dependent variable: family quality of life total score. *B*, unstandardized coefficient; SE, standard error; *β*, standardized coefficient; 95% CI, 95% confidence interval for *B*; sr^2^, squared semipartial correlation (Equation 6); SE_r_ and *p*_r_, heteroscedasticity-consistent (HC3) standard error and its associated *p* value. ASD severity, household income, and educational attainment were entered as ordinal scores; comorbidity coded 0 = none, 1 = present; community support, perceived community support; digital competence, self-rated digital competence. Because the Breusch–Pagan test was significant, inference is based on the SE_r_ and *p*_r_ columns.

Diagnostic checks identified three departures from the assumptions of ordinary least squares. The Breusch–Pagan test was significant, *χ*^2^(8) = 18.46, *p* = 0.018, indicating heteroscedasticity; HC3 standard errors are therefore reported in Table 8 and used throughout. Residuals departed from normality (skewness = −0.85, kurtosis = 0.87; Shapiro–Wilk *W* = 0.956, *p* < 0.001). The Ramsey RESET test was also significant, *F*(2, 283) = 4.66, *p* = 0.010, indicating that the linear specification does not fully capture the relationship between the predictors and FQoL; the consequences are considered in Section 3.6 and in the limitations. Independence of errors was satisfactory (Durbin–Watson = 1.85), and no observation exerted undue influence (maximum Cook’s distance = 0.042).

In the dummy-coded sensitivity model (12 predictors), the fit was essentially unchanged, *F*(12, 281) = 13.98, *p* < 0.001, *R*^2^ = 0.374, adjusted *R*^2^ = 0.347. Full coefficients with HC3 errors and confidence intervals are given in Table 2. Caregiver burden, perceived community support, and comorbidity retained significance and magnitude; digital competence and accessibility remained non-significant. None of the three income contrasts and neither education contrast approached significance (all *p* ≥ 0.410), and the moderate-versus-mild severity contrast fell just short (*B* = 0.056, *p* = 0.055) while the severe-versus-mild contrast was null (*B* = −0.087, *p* = 0.293). The socioeconomic and severity variables therefore carry no reliable association with FQoL under either coding.

### 3.6 Robustness and sensitivity analyses

Floor and ceiling occupancy was nil for every composite: no participant scored at the floor or the ceiling of any scale, so the bounded outcome does not truncate the distribution. Mean FQoL across tertiles of each continuous predictor was monotonic for burden, support, and digital competence, and flat for accessibility. The significant RESET test is therefore not attributable to boundary effects; it more plausibly reflects mild curvature in one or more predictor relationships.

The three alternative estimators agreed with ordinary least squares on every conclusion. Percentile bootstrap intervals from 5,000 resamples excluded zero for perceived community support, caregiver burden, and comorbidity and included zero for the other five predictors—the same pattern as the HC3 intervals. Huber M-estimation and median regression reproduced the sign and approximate magnitude of every coefficient (median-regression pseudo *R*^2^ = 0.210). Full results are given in Table 3.

Although bootstrap, Huber M-estimation, and median regression produced similar signs and inferential conclusions, these analyses address influential observations and error-distribution assumptions and do not directly resolve the significant RESET result, which concerns functional form. The linear model is therefore retained as a pragmatic approximation, and its coefficients should be interpreted as exploratory associations rather than as a validated functional representation of the underlying relationships.

Two modified samples were also examined. Excluding the eight participants in the lowest income band left every coefficient essentially unchanged (*n* = 286, *R*^2^ = 0.362), as did excluding the 10 professional caregivers (*n* = 284, *R*^2^ = 0.361), although in the latter the comorbidity coefficient fell just below the conventional threshold (*B* = −0.061, *p* = 0.056), indicating that this coefficient is not robust to the removal of 10 cases and should be regarded as the least secure of the three significant associations.

## 4 Discussion

This cross-sectional study examined the associations of caregiver burden, perceived community support, self-rated digital competence, and service accessibility with family quality of life among 294 Saudi caregivers of individuals with ASD. The final regression model accounted for 35.7% of the variance in FQoL. That figure describes the proportion of variance the model accounts for within this sample under this specification; it is not an estimate of generalizable explanatory power or of predictive accuracy in a new sample, and it is inflated to an unknown degree by the fact that outcome and predictors were reported by the same person on the same occasion using instruments developed together. Two associations were comparatively consistent across the reported specifications—perceived community support and caregiver burden—whereas the association with comorbidity was weaker and sensitive to the exclusion of professional caregivers. These three primary-model associations are discussed below, with their differing levels of robustness made explicit; each should be read as an exploratory association rather than as an identified determinant.

First, perceived community support was the strongest independent correlate of FQoL (*β* = 0.39) and, on the evidence of Block 4, the only one of the three resource variables to carry an independent association. Because the items concern support obtained through caregiver networks and online communities, the finding is narrower than the general proposition that social support protects family wellbeing (Khusaifan and El Keshky, 2025; Lai et al., 2015; Wang et al., 2021): what this study can say is that caregivers who report receiving more support through these particular channels also report better family quality of life. The direction of that relationship is not identified by the design. Caregivers whose families are functioning well may have more capacity to seek and sustain community connections, and a caregiver disposed to report favorably about family life may also report favorably about the support received.

Second, caregiver burden retained a substantial negative association with FQoL after adjustment for clinical and sociodemographic characteristics (*β* = −0.26), consistent with the structural-equation evidence reported by Wang et al. (2021) and the observations of Marsack-Topolewski and Church (2019). Notably, burden was essentially uncorrelated with perceived community support (*ρ* = 0.06) and digital competence (*ρ* = 0.02) in these data, so its contribution is largely independent of the resource block. Whether interventions that reduce burden would in turn improve FQoL cannot be established from cross-sectional data; this hypothesis warrants testing in longitudinal and experimental designs.

Third, the presence of a comorbid condition was associated with lower FQoL in both the group comparison (*r*_rb_ = 0.37) and the adjusted model (*β* = −0.13). The direction is consistent with the wider literature on the additional demands that co-occurring conditions place on families. It is, however, the least robust of the three associations: it falls just below the conventional threshold when the 10 professional caregivers are excluded (Section 3.6), and it should not be placed on the same evidential footing as the support and burden coefficients.

Three constructs central to the study’s framing carried no independent association. Self-rated digital competence correlated with FQoL at the bivariate level (*ρ* = 0.31) but did not survive adjustment (*p* = 0.098), which is unsurprising given its correlation of 0.57 with perceived community support: the two composites appear to be measuring overlapping aspects of engagement with digital caregiving environments, and when both are entered the support composite absorbs the shared variance. The measure is also the least reliable in the battery (*α* = 0.66), so its coefficient is attenuated by measurement error, and the study is underpowered to resolve an effect of this size. We therefore report this as inconclusive rather than as evidence that digital competence is unrelated to family quality of life.

Service accessibility showed no association with FQoL at any stage—neither bivariately (*ρ* = −0.013) nor after adjustment (*p* = 0.409). We do not interpret this as evidence that access is unimportant to families. The composite is a weak instrument: its internal consistency falls below the conventional threshold (*α* = 0.66), principal-component analysis returns three components with the first accounting for only 31.3% of item variance, and it averages availability, geographic reach, appointment acquisition, and satisfaction with government and private provision into a single score. A null result from a measure with these properties is uninformative about the underlying construct, and the appropriate conclusion is that this study did not measure service accessibility well enough to test its relationship with family quality of life.

Neither household income nor educational attainment nor caregiver-reported ASD severity was independently associated with FQoL. Income showed a bivariate gradient, but no individual band contrast survived dummy coding and the adjusted coefficient was non-significant (*p* = 0.110). Severity differed between the mild and moderate groups but not in an ordered way, and it carried no adjusted association; with only 10 participants in the severe group, the study provides no evidence about severe presentations at all. None of these variables is advanced as a finding.

### 4.1 Limitations

Several limitations warrant acknowledgment. First, the cross-sectional design precludes inferences about temporal precedence or causal direction; reciprocal influences are plausible, and all regression findings should be read as statistical associations.

Second, and most consequentially, all five composites were written for this study and none has been independently validated. Two fall below the internal-consistency threshold the study itself adopts: self-rated digital competence (*α* = 0.66) and service accessibility (*α* = 0.66). More seriously, the five FQoL subscales are not reliable at all (*α* = 0.27–0.46), so the instrument supports only a total score, and the accessibility composite has a marginal component structure, with a single component retained by parallel analysis accounting for only 31.3% of item variance and two of its nine items loading near zero on it. Internal consistency is in any case not construct validity: no expert panel, content-validity index, or forward–back translation procedure was applied, no confirmatory factor analysis in an independent sample is available, and published Arabic instruments that could have been used instead were not administered. The composites should be read as study-specific exploratory indicators; the labels perceived community support and self-rated digital competence were chosen to reflect item content rather than the broader constructs of perceived social support and digital health literacy, and results should not be transferred to those constructs.

Third, 122 of the 416 submitted responses (29.3%) were excluded for missing items on the outcome measure. Missingness on an outcome cannot be assumed unrelated to that outcome, and the analytic sample of 294 may therefore differ systematically from the full set of respondents in ways this study cannot assess. No imputation was attempted, since the missingness falls entirely on the dependent variable.

Fourth, the FQoL measure follows the five Beach Center domains but was administered with frequency rather than satisfaction anchors, so scores are not directly comparable with the international literature using the published scale. Comparisons of absolute levels with prior studies should be avoided.

Fifth, the dispersion of every composite is narrow—the FQoL total spans 2.52 to 4.28 with a standard deviation of 0.30, and the three-point composites span less than one scale point. Restricted variance attenuates correlations and limits the precision of the regression estimates, and it means that coefficients expressed on the original metric describe changes across a range that few participants actually occupy.

Sixth, ASD severity was caregiver-reported, unverified against clinical documentation, and severely imbalanced, with only 10 severe cases. No conclusion about severe presentations can be drawn from these data, and the significant mild-versus-moderate difference is non-monotonic and disappears under adjustment.

Seventh, all constructs were assessed by self-report from a single informant on a single occasion, introducing the possibility of social desirability and common-method bias (Podsakoff et al., 2003). The correlation of 0.57 between perceived community support and self-rated digital competence, and the fact that the former absorbs the latter’s association with FQoL, are consistent with the two composites tapping a common response tendency as well as a common substantive domain.

Eighth, the model residuals were heteroscedastic and non-normal, and the Ramsey RESET test was significant (*p* = 0.010), indicating that the linear specification does not fully capture the relationships in the data. Robust standard errors address the heteroscedasticity, and the alternative estimators in Table 3 reproduce every inferential conclusion, but those estimators bear on influential cases and error distribution rather than on functional form and so do not resolve the RESET result. The linear model is retained as a pragmatic approximation; its coefficients should be read as exploratory associations rather than as a validated functional representation of the underlying relationships.

Ninth, the achieved sample of 294 falls well short of the 463 the a priori calculation required. Non-significant coefficients—self-rated digital competence in particular, at *p* = 0.098—are inconclusive rather than evidence of no association.

Tenth, the survey was distributed and completed entirely online, so caregivers with limited digital access or literacy could not take part and are absent from the sample. This is of particular consequence for a study concerned with digital resources, since the observed associations are estimated only among caregivers already able to use an online platform. The non-probability design compounds this, and no denominator exists from which a response rate could be calculated. The screening check found no duplicate records, but because the platform recorded no timestamps or respondent identifiers, the independence of responses cannot be verified beyond that check.

Eleventh, the unit of analysis is ambiguous. Family quality of life is a family-level construct, but it was reported by one member of each family, and 10 respondents (3.4%) were professional rather than family caregivers, for whom the notion applies less directly. Excluding them leaves the two principal findings intact but renders the comorbidity coefficient non-significant (Section 3.6), so the reported scores are best understood as one caregiver’s appraisal of family functioning rather than a family-level measurement.

Finally, several potentially relevant variables were not measured, including caregiver mental health, adaptive functioning and behavior problems of the family member with ASD, time since diagnosis, and the type and intensity of intervention services received, leaving open the possibility of omitted-variable confounding.

### 4.2 Implications for practice

Only one of the three resource variables carried an independent association with family quality of life, and nothing in the design licenses the inference that strengthening it would raise family quality of life; the association may run in either direction, or arise from a common cause such as the general disposition of a caregiver who reports favorably about several domains at once. What follows is therefore a hypothesis for intervention research, not a demonstrated effect. Facilitating caregivers’ connection to structured peer and online communities, rather than leaving such connection to chance, is worth evaluating experimentally, as is caregiver burden screening using brief validated instruments embedded in routine autism visits with structured referral pathways. The null results for self-rated digital competence and service accessibility should not be read as showing those factors to be unimportant: the first is inconclusive at this sample size and the second was measured with a composite too weak to support any conclusion. Both warrant re-examination with validated instruments before practice implications are drawn either way.

## 5 Conclusion

In this exploratory cross-sectional study, perceived community support and caregiver burden were the most consistent correlates of family quality of life across the reported model specifications. The association with comorbidity was present in the primary model and in the estimator-based robustness checks but was not robust to the exclusion of professional caregivers, and it is therefore reported with less confidence than the other two. Self-rated digital competence, service accessibility, household income, educational attainment, and caregiver-reported severity carried no independent association; the first is inconclusive at this sample size and the second was measured too weakly to be tested. Because the design was cross-sectional, all five composites were developed for this study without independent validation and two fall below conventional reliability thresholds, 29.3% of submitted responses were lost to missing outcome data, and the sample was a non-probability online sample, these results identify hypotheses rather than determinants. The priorities for further work are validation of the measures against established instruments, replication with clinician-assigned severity and a validated access measure, and longitudinal or intervention designs capable of establishing directionality.

## Data Availability

The de-identified dataset and the complete analysis script that produced every value reported in this article are provided as Supplementary material. The script reproduces all tables, figures, and reported statistics in a single run from the raw data file, including the duplicate-record audit and every sensitivity analysis reported in Section 3.6. Further enquiries may be directed to the corresponding author.
