# Supplementary material for: Caregiver burden and perceived community support as correlates of family quality of life among caregivers of individuals with autism spectrum disorder in Saudi Arabia
Source: Front Psychol. 2026 Sep 30;17:1897617. doi: 10.3389/fpsyg.2026.1897617 (PMC13661551; doi:10.3389/fpsyg.2026.1897617)
Supplement: Supplementary file 1 [file Supplementary_file_1.pdf]

Supplementary Table S1. **Item-level completeness of the comparison variables (all 416 rows)**

Variable	Number of items	Rows with any item missing / missing values
Caregiver burden items	31	0
Perceived support items	44	0
Digital competence items	35	0
Service accessibility items	9	0
Caregiver role	â€”	0
Educational attainment	â€”	0
Household income	â€”	0
Region of residence	â€”	0
ASD severity	â€”	0
Comorbid condition	â€”	0

Note. Composite variables were defined listwise, consistent with the manuscript definition. The analytic sample comprised 294 respondents with complete Family Quality of Life (FQoL) items; 122 responses were excluded.

Supplementary Table S2. **Comparison of the analytic sample and excluded responses**

Variable	Included (n = 294)	Excluded (n = 122)	Test	Statistic	p	Effect size	95% CI	Interpretation
Caregiver burden	2.25 (0.23)	2.26 (0.20)	Mannâ€“Whitney U	18,054.0	.915	.01	[0.00, 0.11]	No evidence of difference
Perceived community support	2.37 (0.19)	2.40 (0.17)	Mannâ€“Whitney U	16,098.5	.100	.08	[0.01, 0.18]	No evidence of difference
Self-rated digital competence	2.38 (0.15)	2.39 (0.14)	Mannâ€“Whitney U	16,919.0	.363	.04	[0.00, 0.14]	No evidence of difference
Service accessibility	3.69 (0.45)	3.67 (0.44)	Mannâ€“Whitney U	18,568.5	.570	.03	[0.00, 0.13]	No evidence of difference
Caregiver role	â€”	â€”	Ï‡Â²(4)	7.70	.103	.14	[0.08, 0.24]	No evidence of difference
Educational attainment	â€”	â€”	Ï‡Â²(2)	2.39	.303	.08	[0.02, 0.19]	No evidence of difference
Household income	â€”	â€”	Ï‡Â²(3)	2.14	.544	.07	[0.03, 0.18]	No evidence of difference
Region of residence	â€”	â€”	Ï‡Â²(4)	2.61	.626	.08	[0.05, 0.20]	No evidence of difference
ASD severity	â€”	â€”	Ï‡Â²(2)	0.38	.827	.03	[0.01, 0.14]	No evidence of difference
Comorbid condition	â€”	â€”	Ï‡Â²(3)	1.36	.716	.06	[0.03, 0.18]	No evidence of difference

Note. Values for continuous variables are mean (standard deviation). MW U = Mannâ€“Whitney U. For Mannâ€“Whitney U tests, the effect size is r = |z|/âˆšN. For chi-square tests, the effect size is Cramerâ€™s V. The 95% confidence intervals are percentile bootstrap intervals derived from 5,000 resamples.

Supplementary Table S3. **Parallel analysis for service-accessibility items**

Component	Observed eigenvalue	Random 95th percentile	Retain?
1	2.82	1.36	Yes
2	1.11	1.24	No
3	1.08	1.17	No
4	0.99	1.09	No
5	0.78	1.03	No
6	0.71	0.97	No
7	0.57	0.92	No
8	0.49	0.87	No
9	0.45	0.80	No

Note. Parallel analysis used the 95th-percentile criterion with 1,000 simulated datasets (n = 294; nine items). One component was retained. The first component explained 31.3% of the total variance.

Supplementary Table S4. **Unrotated loadings on the first three principal components**

Item	PC1	PC2	PC3
Availability in area	0.00	âˆ’0.48	0.24
Ease of reaching specialists	âˆ’0.15	âˆ’0.15	0.82
Satisfaction, government	0.60	âˆ’0.46	0.00
Satisfaction, private	0.69	âˆ’0.32	0.09
Medical appointment	0.57	0.12	âˆ’0.38
Diagnostic appointment	0.60	0.48	0.22
Educational/rehabilitation appointment	0.50	âˆ’0.43	âˆ’0.27
Consultative appointment	0.70	0.27	0.24
Social services appointment	0.74	0.19	0.15

Note. PC = principal component. The parallel-analysis result supports a one-component solution for the service-accessibility scale.
